# Nutrition of the COVID-19 patient in the intensive care unit (ICU): a practical guidance

**DOI:** 10.1186/s13054-020-03159-z

**Published:** 2020-07-19

**Authors:** Ronan Thibault, Philippe Seguin, Fabienne Tamion, Claude Pichard, Pierre Singer

**Affiliations:** 1 Unité de Nutrition, CHU Rennes, 2, rue Henri Le Guilloux, 35000 Rennes, France; 2 INRAE, INSERM, Nutrition Métabolismes et Cancer, NuMeCan, Univ Rennes, Rennes, France; 3 Service de Réanimation chirurgicale, CHU Rennes, Rennes, France; 4 Service de Réanimation Médicale, Normandie Univ, UNIROUEN, U1096, CHU de Rouen, F 76000 Rouen, France; 5 Unité de Nutrition, Hôpitaux universitaires de Genève, Geneva, Switzerland; 6 Department of General Intensive Care and Institute for Nutrition Research, Rabin Medical Center, Beilinson Hospital, Sackler School of Medicine, Tel Aviv University, Tel Aviv, Israel

**Keywords:** Malnutrition, SARS-Cov2 virus, Critical illness, Enteral nutrition, Supplemental parenteral nutrition, Energy target, Protein target

## Abstract

Five to 10% of the coronavirus SARS-CoV-2-infected patients, i.e., with new coronavirus disease 2019 (COVID-19), are presenting with an acute respiratory distress syndrome (ARDS) requiring urgent respiratory and hemodynamic support in the intensive care unit (ICU). However, nutrition is an important element of care. The nutritional assessment and the early nutritional care management of COVID-19 patients must be integrated into the overall therapeutic strategy. The international recommendations on nutrition in the ICU should be followed. Some specific issues about the nutrition of the COVID-19 patients in the ICU should be emphasized. We propose a flow chart and ten key issues for optimizing the nutrition management of COVID-19 patients in the ICU.

## Introduction

The viral epidemic caused by the new coronavirus SARS-CoV-2 is responsible for the new coronavirus disease 2019 (COVID-19) [1]. Up to 30% of the coronavirus SARS-CoV-2-infected patients are presenting with an acute respiratory distress syndrome (ARDS) requiring urgent respiratory and hemodynamic support in the intensive care unit (ICU) [2]. The coronavirus SARS-CoV-2 is colonizing the respiratory tract but may also invade the gastrointestinal (GI) tract [3–10], neurological system, and kidneys [11]. SARS-CoV-2 uses the angiotensin-converting enzyme 2 receptor as an entry receptor in the lymphocytes, monocytes, lung alveolar type 2 cells, esophagus epithelial cells, enterocytes, and colonocytes [12], creating rapid viral replication and cell damage that induce huge inflammation and increased cytokine secretion. In the most severe cases, it leads to a cytokine storm with high proinflammatory cytokine plasma levels [2]. Lung histopathological changes are compatible with diffuse alveolar damage. This damage is often lethal. The primacy of the resuscitation measures should not obscure the importance of nutritional care.

The length of time for recovery for patients who survive COVID-19 is a key factor that nutrition is vital for. ICU survivors are staying for long periods [13]. In Seattle, survivor patients were ventilated for 10 days (mean) and stay in hospital for 18 days [14]. In Lombardia [15], from 1591 patients requiring ICU, the median (IQR) ICU length of stay was 9 (6–13) days. Therefore, it is expected that COVID-19 patients who survived ICU would present severe malnutrition and muscle mass loss.

The nutritional assessment and the early nutritional care management of COVID-19 patients must be integrated into the overall therapeutic strategy, as with any critical illness and rehabilitation program. As there is a COVID-19 GI and liver involvement [3–10], it may have an effect on nutrition delivery. This review is intended to help ICU health professionals to optimize nutrition management of COVID-19 patients, especially those with ARDS. This article was written in the emergency of the epidemic by an expert group, based on the international recommendations on nutrition in the ICU on March 29, and will be updated according to new knowledge about the COVID-19.

## Nutritional consequences of COVID-19 disease

The COVID-19 patients with the most severe forms as seen in the ICU are more frequently elderly and with comorbidities [16] and therefore at major risk of malnutrition and sarcopenia [17, 18]. In the absence of nutritional data specific to COVID-19, the following considerations are proposed from the data related to severe respiratory infections:

- Severe respiratory infections induce inflammatory syndrome and hypercatabolism, with increased energy expenditure linked to ventilatory work, in turn responsible for increased energy and protein requirements;
- Food intake is very reduced by several factors: anorexia secondary to infection, dyspnea, dysosmia, dysgeusia, stress, confinement, and organizational problems limiting attendance at meals. Most COVID-19 patients admitted to the ICU are at high risk of malnutrition;
- Infection, hypermetabolism, and physical immobilization expose to rapid muscle wasting. The worsening of malnutrition should therefore be prevented by an appropriate nutritional strategy, including adequate protein-energy delivery and stimulation of physical activity.

## Practical guidance of the nutritional treatment of the patient with COVID-19 in the ICU

**Fig. 1:**
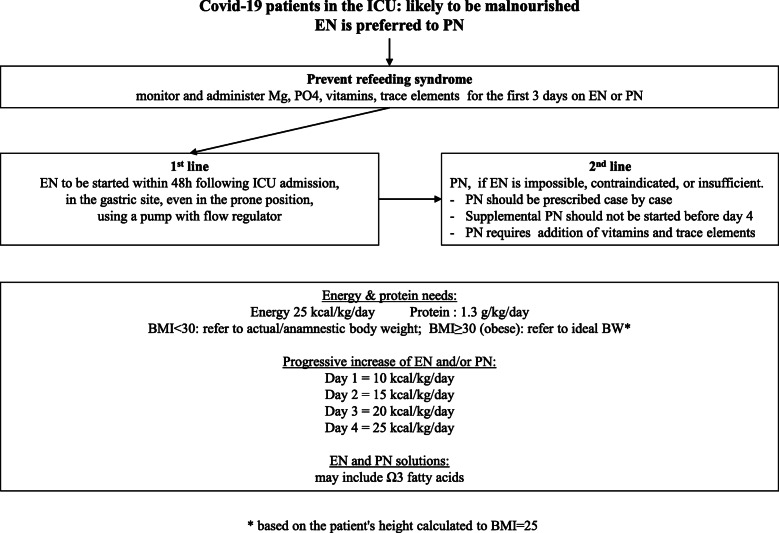
Nutrition support protocol for the patient with COVID-19 in the intensive care unit. BMI, body mass index; BW, body weight; EN, enteral nutrition; ICU, intensive care unit; IV, intravenous; PN, parenteral nutrition

Based on the recommendations of the European Society for Clinical Nutrition and Metabolism (ESPEN) on nutrition in the ICU [18], a COVID-19 patient’s nutrition protocol is proposed in Fig. 1. The main specificities are listed below and then further discussed:

1. COVID-19 patients should be considered for malnutrition.
2. Nutritional evaluation based on the Global Leadership Initiative on Malnutrition (GLIM) [19] should be adapted to the COVID-19 epidemic.
3. Indirect calorimetry should be proposed only for patients staying for more than 10 days in the ICU or those on full parenteral nutrition (PN) to avoid overfeeding.
4. Refeeding syndrome (RS) [18, 20, 21] and complications related to propofol use must be prevented.
5. Enteral nutrition (EN) should be preferred over PN and started within 48 h of admission.
6. Gastric EN is generally possible, including in the prone position, and should be preferably performed using a pump with flow regulator.
7. PN is indicated if EN is impossible, contraindicated, or insufficient and should be prescribed using a case-by-case decision making.
8. The use of EN enriched with omega-3 fatty acids should be preferred in case of ARDS. Fish oil-enriched intravenous fat emulsions should be prescribed if PN is required.
9. After extubation, the nutritional support is promoting patient’s recovery and rehabilitation and should be continued until the patient resumes sufficient oral intake.
10. Physical activity should be promoted to preserve muscle mass and function.

### COVID-19 patients should be considered for malnutrition

COVID-19 is a disease at high risk of malnutrition. The most severe cases are encountered in particular, but not exclusively, in patients with a chronic disease (such as organ failure, obesity with body mass index ≥ 40, type 2 diabetes or cancers), who are elderly, and/or with polypathologies [2, 16]. These diseases often mask underlying protein malnutrition (sarcopenia). Malnutrition is a factor of poor prognosis and should therefore be actively investigated, even in the absence of specific literature concerning COVID-19.

### Nutritional evaluation based on the Global Leadership Initiative on Malnutrition (GLIM) [19] should be adapted to the COVID-19 epidemic

The 2018 international consensus for malnutrition diagnosis by GLIM defined new criteria for the diagnosis of malnutrition [19]. According to the GLIM criteria, a patient is malnourished if he/she has at least one phenotypic criterion and at least one etiologic criterion. Phenotypic criteria are body mass index < 20 (or < 22 if age ≥ 70 years) or weight loss > 5% within past 6 months or > 10% beyond 6 months or reduced muscle mass; etiologic criteria are reduced food intake (≤ 50% in > 1 week or reduced food assimilation (malabsorption or previous history of GI surgery) or acute disease/injury/chronic disease-related inflammation.

In the context of the COVID-19 epidemic, the phenotypic criteria are poorly applicable:

- Because of the risk of increased viral transmission by patient contacts, weight machine and height chart are difficult to use since they must be decontaminated after each use. ICU beds with integrated weight system define body weight difficult to interpret due to the fluid overload, especially in case of severe hypoalbuminemia or shock resuscitation.
- Similarly, bioelectrical impedance analysis and measurement of muscle strength by handgrip dynamometry are not recommended in COVID-19 patients due to the risk of increased viral transmission by patient contacts.

Therefore, the nutritional screening at admission should be based on:

- Patient’s or relatives’ interview to determine recent weight loss before admission and body mass index. If possible, food intake may also be quickly and easily assessed with semi-quantitative methods: an analogue scale between 0 and 10/10 [22] or consumed portions (0, ¼, ½; 1) during the last lunch or dinner, as did in the NutritionDay survey [23]; taking < 7/10 should alert to likely malnutrition [22].

Moreover, two etiologic criteria for malnutrition diagnosis (according to GLIM recommendations [19]) are obvious in the COVID-19 patients:

- COVID-19, as an “acute disease,” is associated with acute inflammation.
- According to the more recent data [24], hypoalbuminemia is associated with a worse prognosis of COVID-19.

### Indirect calorimetry (IC) should be proposed only for patients staying for more than 10 days in the ICU or those on full parenteral nutrition (PN) to avoid overfeeding

IC is the reference method to assess the energy requirements in the non-COVID-19 ICU patients [18]. However, in the context of the COVID-19 epidemic, but depending on location, some ICUs experience massive overload of COVID-19 patients. That context precludes the performance of any sophisticated non-vital methods at the early phase of ICU stay. Moreover, there is still an uncertainty regarding the safe use of IC, as the usual decontamination procedures cannot be guaranteed in an epidemic context. Therefore, committed IC devices and virus filters for COVID-19 patients only should be considered when possible. To avoid exposure to aerosol and potential virus contamination during IC device connection/disconnection, our recommendations are, previous to connection to the IC device, to put the ventilator on standby and to clamp the tube, then connect and when connected to declamp the tube and to restart the ventilator. This way is preventing the potential spread of virus during disconnection/connection.

Therefore, in these conditions, we propose that IC should be performed to all patients after 3–4 days in the ICU. Depending on staff organization and material availability, patients staying longer than 10 days in the ICU or those on full PN should be the priority. Indeed, the patients on full PN are the most at risk of developing the serious complications related to overfeeding (hyperglycemia, hypertriglyceridemia, bacteremia, liver injury).

Alternatively, to determine calorie and protein needs, we propose the use of predictive equations according to weight (Fig. 1). Introduction of nutrition support should be phased according to day (Fig. 1). The ultimate goal is to avoid underfeeding or overfeeding.

Importantly, obesity is associated with severe forms of COVID-19. In the ICU, obesity is also associated with increased protein catabolism as compared with non-obese patients [25]. It is therefore even more necessary to avoid restrictive and hypocaloric nutrition in obese patients. Underfeeding is more likely in obese patients: obese patients often have increased energy expenditure compared to non-obese [20]; initiation of nutritional support is often delayed in obese ICU patients [21]. In obese ICU patients, rapid weight loss would be associated with increased loss in muscle mass, weakening the immune defenses and therefore promoting COVID-19 severity.

### Refeeding syndrome (RS) [18, 26, 27] and complications related to propofol use must be prevented

The RS is underestimated at ICU admission [28]. COVID-19 patients are often vulnerable (old, polymorbid, malnourished, sarcopenic) [29] and frequently unwell for 9–15 days [13, 30], i.e., presenting fever, asthenia, lack of appetite, reduced food intake, leading to energy deficit before their ICU admission. These characteristics promote the risks of electrolyte imbalances (i.e., refeeding syndrome). We propose to detect/prevent the RS in older patients, those with polymorbidity, no/low food intake for > 5 days, preexisting malnutrition, and abnormal electrolytes due to diuretic treatment and dialysis. Plasma potassium, phosphorus, and magnesium should be measured within 6 h after nutrition support is started to detect and treat low values. For refeeding guideline, please refer to the UK National Institute for Health and Care Excellence (NICE) guidelines [31] https://www.evidence.nhs.uk/search?pa=2&q=refeeding+syndrome.

Specific advices regarding patients under propofol should be stated here. All the sedative drugs, including propofol and benzodiazepines, have immunosuppressive effects [32]. The “propofol infusion syndrome” (PRIS) is a rare complication observed when propofol is used for > 48 h and at high doses (> 4 mg/kg/h) and must be evoked in case of hemodynamic degradation or lactic acidosis without any other causative factor. The monitoring every 72 h of arterial gazometry, plasma lactate, creatine phosphokinase, and triglycerides allows anticipating the risk of PRIS and stop propofol. In the COVID-19, the cytokine storm could lead to hemophagocytosis that could itself increase plasma triglyceride, independently from propofol use. Plasma triglyceride monitoring at least every 72 h is advised in all ICU COVID-19 patients. Moreover, propofol doses should be controlled and alternative sedation should be used if the doses are too high or the treatment is prolonged. Large administration of omega-6 fatty acids is not recommended in the context of strong inflammatory response to virus load.

### Enteral nutrition (EN) should be preferred over parenteral nutrition (PN) and started within 48 h of admission

As usually recommended in the ICU [18, 33], EN should be preferred over PN. A lack of consensus among international academic societies exists about the best timing to start nutrition support after ICU admission. The main reason is the heterogeneity of the ICU patients (age, severity of disease, medical versus surgical cares, preexisting malnutrition, chronic diseases). All over the world, ICU COVID-19 patients are rather similar in terms of vulnerability (older, chronic diseases, low food intake for 5–10 days) [2, 16] and likelihood of prolonged ICU stay on mechanical ventilation [14, 15]. These characteristics support the indication for early (< 48 h after admission) and progressive increase of nutrition support (usually enteral) to reach an energy target by day 4–6 days, depending on tolerance (Fig. 1). This is in line with the main available international guidelines for nutrition during critical illness and COVID-19 [27] https://www.nutritioncare.org/uploadedFiles/Documents/Guidelines_and_Clinical_Resources/Nutrition%20Therapy%20COVID-19_SCCM-ASPEN.pdf, https://www.auspen.org.au/auspen-news/2020/4/6/covid-19-information. The polymeric standard EN formulas should be used like in other ICU patients. If polymeric EN administration is associated with diarrhea, semi-elemental EN can be tested as second line. To our knowledge, there is no specific indication for arginine in ICU COVID-19 patients. As a meta-analysis [34] reported that arginine increases mortality in sepsis and pneumonia patients, arginine should not be used in COVID-19 patients.

### Gastric EN is generally possible, including in the prone position, and should be preferably performed using a pump with flow regulator

In the context of ARDS, EN is frequently delivered in the prone position. This is associated with an increased risk of gastroparesis and vomiting. All should be done to optimize EN (Fig. 1). In the context of the use of hydroxychloroquine associated with azithromycin as an antibiotic therapy against the SARS-CoV2 virus, the preferable prokinetics is metoclopramide, to avoid any drug side effect and interferences. EN during prone position has been shown to be safe in terms of large gastric residue, vomiting, or intolerance [35]. The prone position per se does not represent a limitation or contraindication for EN and is recommended by the ESPEN guidelines [18].

According to a meta-analysis [8], 17% of the patients with severe COVID-19 had GI symptoms (95% CI, 6.9–36.7%), in line with other findings [3–7]. In the meta-analysis, stool samples were positive for SARS-CoV-2 virus DNA in 48.1% of the cases (95% CI, 38.3–57.9%) [8]. However, there is no evidence that COVID-19 patients with recent history of diarrhea, abdominal pain, nausea, and vomiting should be contraindicated to EN. In case of EN intolerance, nutrition delivery should be adapted as described in this section and the “PN is indicated if EN is impossible, contraindicated, or insufficient and should be prescribed using a case-by-case decision making” section. According to the severity of GI symptoms, EN should be temporarily stopped or reduced or combined/switched to supplemental or total PN.

We do not advise the systematic measurement of gastric residual volumes (GRV). In ventilated patients, not measuring GRV was not associated with an increased risk of ventilator-associated pneumonia [36]. ASPEN (https://www.nutritioncare.org/uploadedFiles/Documents/Guidelines_and_Clinical_Resources/Nutrition%20Therapy%20COVID-19_SCCM-ASPEN.pdf) and AuSPEN (https://www.auspen.org.au/auspen-news/2020/4/6/covid-19-information) experts are advising measuring GRV for all prone patients, those under vasopressors or with GI COVID-19. However, there is no clear evidence to support this. Therefore, as viral load may be present in gastric contents, the number of GRV measurements should be reduced. GRV may not be measured in those patients with stable hemodynamic or low dose of vasopressors, including those in the prone position. For others, the decisions should be made case by case, in accordance with the usual ICU department protocols.

#### Safety and prevention of aerosol generating procedures (AGP)

Feeding tube placement and GRV measurement are AGP. Gastric content and stools can contaminate health professionals. Therefore, introduction of nasogastric or postpyloric tube, GRV measurement, or handling of stools should be made very cautiously according to strict protection protocols. In ventilated patients with insufficient sedation, it could happen that patients are agitated, coughing, or vomiting during the tube placement procedure. These patients should be better sedated or paralyzed before the feeding tube placement.

In case gastric EN is complicated with vomiting or gastroparesis, jejunal EN may be considered. However, the nasojejunal tube placement is very challenging as it is an AGP. Therefore, any procedure requiring transfer to radiology suites or endoscopy should be avoided. Only bedside introduction of nasoduodenal or nasojejunal tubes may be recommended, but they are not always available or successful. We may rather recommend the use of PN in case EN is not tolerated (see the “PN is indicated if EN is impossible, contraindicated, or insufficient and should be prescribed using a case-by-case decision making” section).

EN should be performed as much as possible using a pump with flow regulator. In the event of a shortage of pumps with flow regulator, it is necessary to reserve them as a priority for the ICUs. In non-intubated patients, it seems preferable to not use EN rather than doing it without pumps with flow regulator, because of the risk of aspiration pneumonia. Indeed, underfeeding is likely to have less severe consequences than aspiration pneumonia.

#### Feeding on vasopressors and in paralyzed patients

A recent retrospective study has shown that early EN in paralyzed patients was associated with less hospital mortality, and there is no increase in ventilator-associated pneumonia [37]. However, in this study, paralysis duration was short: 48 h. In COVID-19 patients, paralysis is usually longer. There is no data about how to feed the ICU COVID-19 patients who are paralyzed for > 48 h. In our experience, there is no increase in EN complications in these patients. Therefore, we propose to adapt nutrition support as generally done in case of GI intolerance (see the “PN is indicated if EN is impossible, contraindicated, or insufficient and should be prescribed using a case-by-case decision making” section).

As there is no data about feeding under vasopressors in the ICU COVID-19 patients, we propose to refer to a recent review [38] and follow the ESPEN recommendations [18]: “the use of concomitant vasopressors (especially with stable or decreasing doses) should not preclude a trial of EN […]. In very unstable patients, EN may not have priority.”

### PN is indicated if EN is impossible, contraindicated, or insufficient and should be prescribed using a case-by-case decision making

As usually recommended in the ICU [18, 33], PN is indicated whenever EN is impossible or contraindicated or in addition to EN as long as it is insufficient (supplemental PN).

Many patients are still receiving high-flow nasal cannula (HFNC) therapy or non-invasive ventilation in many centers [39, 40]. From a practical point of view, based on the Chinese experience [39], these patients are almost not fed orally or enterally. Therefore, we could advocate the use of PN arguing that “being fed by PN” is better than “being not fed.” As most severe patients have a central line, PN should be administered through a central venous line. In case there are too many drugs on the central line, peripheral PN could be used.

In the specific context of COVID-19, the use of supplemental PN could be advocated if EN in the prone position is associated with vomiting, in case of severe hypoxemia (PaO_2_/FiO_2_ < 50 mmHg with FiO_2_ > 80%), or in general, in the situation when the gut is not functioning [18, 41] (Fig. 1). In that context, supplemental PN should not be started before day 4 [18].

As a general principle and as the COVID-19 is a new and unknown disease, we also advocate that PN would be prescribed using a case-by-case decision making, always having in mind that PN is at high risk of overfeeding and hyperglycemia over 10 mmol/l that must be avoided [18, 42]. Mild-to-moderate liver injury, including elevated amino transferases, hypoproteinemia, and prothrombin time prolongation, is a sign of severe COVID-19 [10]. Liver function tests should be monitored like in any other ICU patients receiving EN or PN.

### The use of EN enriched with omega-3 fatty acids should be preferred in case of ARDS. Fish oil-enriched intravenous fat emulsions should be prescribed if PN is required

A systematic review and meta-analysis on fish oil (FO) enteral supplementation suggests an advantage for eicosapentaenoic acid (EPA) and docosahexaenoic acid (DHA) acid supplementation in ARDS patients in terms of length of ventilation and length of ICU stay, but not mortality [43]. However, these conclusions were based on low-quality studies. A Cochrane analysis found that enteral EPA and DHA may improve oxygenation and length of ventilation and length of stay, but these findings were mainly based on low-quality evidence studies [44]. Negative outcome associated with administration of enteral FO has been only observed when administered in a bolus and with a low protein regimen [45]. The immunoregulator effects of EPA and DHA may have a beneficial impact in the severe cytokine storm observed in SARS-CoV-2 ARDS. Therefore, we suggest that EN enriched with 3.5 g/day EPA and DHA can be administered in this disease, not in a bolus. Higher amounts up to 9 g/day have been administered safely [43].

FO-based intravenous lipid emulsions have been extensively analyzed in a meta-analysis [46] including 49 prospective randomized controlled studies with intervention groups receiving omega-3 fatty acids compared to standard intravenous lipid emulsions (ILEs), as a part of PN covering > 70% of the energy provision. Mortality was not decreased significantly, but a significant decrease was observed in relative risk of infection (40% lower) with omega-3 fatty acid-enriched ILEs, in ICU and in hospital lengths of stay. Risk of sepsis was also reduced by 56%. This latest analysis increases our knowledge on FO-enriched lipid emulsions. If PN including ILEs is required in this population suffering from COVID-19 ARDS, FO-enriched lipid emulsions should be prescribed. The provision of omega-3 fatty acids increases the EPA and DHA plasma levels [47]. The recommended FO doses are 0.1–0.2 g/kg/day.

### After extubation, the nutritional support is promoting patient’s recovery and rehabilitation and should be continued until the patient resumes sufficient oral intake

After extubation, the nutritional strategy must be adapted according to certain situations. After a median of 28 days at hospital [13–15, 30], patients are likely to be malnourished. In most patients, EN should be continued as patients are transitioned to oral diet but not sufficiently to cover their protein-energy needs. This is critical to enhance COVID-19 recovery. Post-extubation swallowing disorders are frequent—10 to 67% of patients [17]—and at risk of insufficient oral intake, therefore malnutrition. After extubation, approximately 24% of elderly patients require EN in addition to their oral food intake [18]. In the context of the COVID-19 epidemic, this proportion would be expected to be higher, due to prolonged resuscitation and the intensity of the inflammatory and catabolic syndrome.

Based on the ESPEN recommendations [18], we propose the following:

- In any situation: in case of dysphagia, provide a diet with a suitable texture. The energy and protein intake must be adapted to the needs. A suitable physical activity or muscle strengthening exercising must be offered.
- In case of post-extubation swallowing disorders: continue EN but assess the risk of aspiration pneumonia. If there is, try to carry out EN at the post-pyloric site. If EN is not possible (e.g., if swallowing rehabilitation may require removal of the feeding tube), propose a temporary PN.
- In case of tracheostomy: favor oral, fractional, enriched feeding with oral nutritional supplements. If the energy and protein needs are not covered (< 70% of the needs), consider supplemental PN by avoiding overnutrition and hyperglycemia > 10 mmol/l.

### Physical activity should be promoted to preserve muscle mass and function

Depending on the individual clinical condition, mobilization at bedside will be encouraged to preserve muscle reserves and function and enhance recovery. It will be adapted to the patient’s capacity for autonomy, in a context of limited availability and access by physiotherapists for priority respiratory care. Mobilization will be intensified as soon as the clinical improvement allows.

## Conclusion

Optimized nutrition care of the ICU COVID-19 patients is important to maintain GI tract function, sustain immune defenses, and avoid severe loss of muscle mass and function. As for any other ICU patients, the latter is crucial to promote short- and long-term recovery. Dedicated studies about the nutrition in the COVID-19 patients are now awaited to enrich our knowledge about the metabolism of this new disease and adapt the nutrition support strategy.

## Data Availability

Not applicable
